# Risk Factors for the Development of Olecranon Bursitis—A Large-Scale Population-Based Study

**DOI:** 10.3390/jcm13247801

**Published:** 2024-12-20

**Authors:** Shai Shemesh, Ron Itzikovitch, Ran Atzmon, Assaf Kadar

**Affiliations:** 1 Department of Orthopaedic Surgery, Samson Assuta Ashdod University Hospital, 7 Ha’Refua Street, Ashdod 77476, Israel; ranat@assuta.co.il; 2 Faculty of Health Sciences, Ben Gurion University, Beer-Sheva 8410501, Israel; 3 Independent Researcher, Kfar Saba 4424309, Israel; 4 Roth|McFarlane Hand & Upper Limb Centre, St. Joseph’s Hospital and Western University, London, ON N6A 4V2, Canada; assaf.kadar@sjhc.london.on.ca

**Keywords:** olecranon bursitis, diabetes mellitus, hyperlipidemia, statins

## Abstract

**Background:** Olecranon bursitis (OB) involves fluid accumulation in the bursa, with common causes being trauma and preexisting conditions. Its incidence is difficult to quantify, and risk factors such as diabetes, obesity, and male gender are frequently noted. Hyperlipidemia has been linked to musculoskeletal disorders, but its role as a risk factor for OB remains unexplored. This study aimed to investigate the association between OB and hyperlipidemia, diabetes, obesity, cardiovascular disease, and statin use. **Methods:** A retrospective cohort study analyzed a large-scale database (2005–2020), ultimately including 10,301 patients with olecranon bursitis and 44,608 controls after applying exclusion criteria. Participants were aged 18–90 years, with BMI between 10 and 55. Key variables such as smoking, diabetes, hyperlipidemia, statin use, cardiovascular diseases (CVDs), and cerebrovascular accidents (CVAs) were analyzed. Logistic regression models were applied with stabilized inverse probability of treatment weighting (IPTW) to estimate odds ratios (ORs) for risk factors, and *p*-values were adjusted using the Benjamini–Hochberg method. **Results:** OB was significantly associated with male gender (OR: 1.406; *p* < 0.0001), hyperlipidemia (OR: 1.239; *p* < 0.0001), statin use (OR: 1.117; *p* = 0.0035), and smoking (OR: 1.068; *p* = 0.0094). Age and BMI were significant continuous variables influencing OB risk, particularly in older patients and those with elevated BMI. CVDs and diabetes were not significantly linked to OB. Hyperlipidemia increased OB risk, especially in males and individuals with higher BMI. **Conclusions:** Male gender, hyperlipidemia, and smoking are key risk factors for OB, with hyperlipidemia posing a notable risk in older individuals and those with higher BMI. Statin use did not significantly alter OB risk in hyperlipidemic patients. Further studies are needed to clarify the mechanisms behind these associations.

## 1. Introduction

Olecranon bursitis (OB) is characterized by fluid accumulation in the bursa with or without inflammation. It is relatively common, with a reported minimum annual incidence of up to 10 per 100,000 [1]. However, exact incidence remains uncertain and difficult to quantify [2], likely being underestimated as mildly symptomatic, healthy individuals may not seek medical care [3].

Two main causes of OB are trauma and preexisting medical conditions, leading to the classification of OB as either traumatic or idiopathic [3]. However, it can also be classified as acute, chronic, or septic [2]. Idiopathic bursitis is one that usually develops in the presence of a predisposing condition, including crystalline disease, diabetes, alcoholism, chronic renal insufficiency, psoriasis, immunodeficiency, intravenous drug use, and obesity [1,3,4,5]. Male predominance is a consistent finding in the literature [2,6,7,8]. The most common physical examination findings were tenderness, erythema/cellulitis, warmth, report of trauma or evidence of a skin lesion, and fever [2].

Treatment options for non-septic olecranon bursitis include compression bandaging with nonsteroidal anti-inflammatory drugs (NSAIDs), needle aspiration of the bursal fluid, needle aspiration with steroid injection, or surgical management such as open or arthroscopic bursectomy, osseous resection, and percutaneous suction drainage [1,4,9,10,11].

In recent years, an association has been identified between various systemic metabolic disorders and distinct musculoskeletal pathologies. Specifically, the correlation between hyperlipidemia, a disorder marked by abnormal blood lipid levels significantly impacting the vascular system and internal organs [12], and tendon-related injuries, such as adhesive capsulitis [12,13] and rotator cuff disease [12,14], has been actively investigated. Although the underlying pathophysiological mechanism remains incompletely elucidated, one proposed hypothesis is that tendon degeneration in hyperlipidemic states arises from the accumulation of cholesterol byproducts, which induce structural and biomechanical alterations within the tendon matrix [15,16,17]. To our knowledge, no epidemiological studies have investigated hyperlipidemia as a risk factor for OB or examined the potential impact of statin use on OB risk in patients with hyperlipidemia.

The purpose of the following longitudinal population-based study was to investigate the association between OB and several conditions: hyperlipidemia and statin use, diabetes mellitus, obesity, and cardiovascular and cerebrovascular diseases.

## 2. Methods

A population-based cohort spanning a 15-year period, from 2005 to 2020, from the Clalit Health Services database was used for this retrospective study. Clalit Health Services is the largest health provider and insurer in Israel, insuring over 65% of the population [18]. Following approval from the institutional review board, a comprehensive examination was executed on the Clalit Health Services database to isolate patients with a confirmed diagnosis of OB. This yielded a total of 100,461 identifiable subjects. Simultaneously, a control group was constructed, comprising 491,977 randomly selected individuals from the broader population within the Clalit Health Services database. None of these individuals had a previously documented diagnosis of OB or any record of OB at any point in their lifetime up to 2020. Notably, the diagnosis was determined solely based on International Classification of Diseases (ICD-9) codes. The database did not provide information regarding the specific etiology of OB, including whether it was septic or non-septic, nor did it indicate whether the condition was trauma-related.

**Figure 1:**
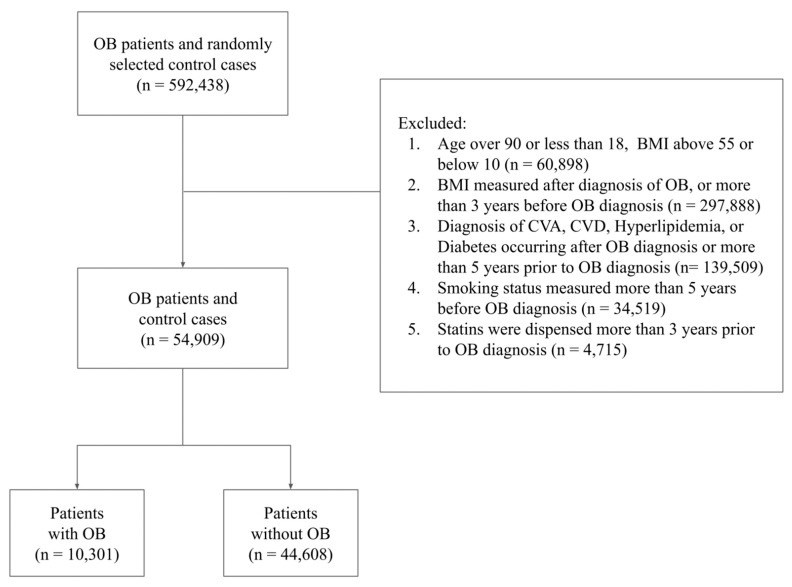
Flow chart of study participants describing the exclusion process and final study population.

The aggregate population for this research, encapsulating both groups, reached 592,438 subjects. Following this, an evaluation was undertaken to ascertain eligibility for inclusion in the final study cohort, in line with the following criteria: Subjects must be aged between 18 and 90 years, with a Body Mass Index (BMI) ranging from 10 to 55. In the case of subjects afflicted with OB, BMI measurements must have been captured within the three years preceding the diagnosis. If subjects possessed any pre-existing medical conditions and were subsequently diagnosed with OB, these comorbidities must have been recognized and documented within a five-year timeframe preceding the OB diagnosis. The conditions considered included smoking status, CVA, CVD, hyperlipidemia, and diabetes mellitus. For subjects diagnosed with OB, statin use must have been recorded within three years preceding the diagnosis. The process of data filtering used to derive the final study population is comprehensively represented in Figure 1.

For the purpose of practical analytical comprehensibility, both age and Body Mass Index (BMI) were treated variably, either as continuous variables in some sections of the analysis, or as categorical in others. In the latter approach, age was grouped into five distinct brackets, namely, 18–44, 45–54, 55–64, 65–74, and 75–90 years, while BMI was delineated into five ranges: 10–18.4, 18.5–24, 25–29, 30–34, and 35–55.

Smoking status was identified as a binary variable (Yes/No), denoting whether an individual was a smoker in the five-year period leading up to their OB diagnosis. Similarly, medical histories concerning hyperlipidemia, CVA, CVD, diabetes mellitus, and statin use were represented as binary variables (Yes/No).

In order to maintain the clarity and reliability of our research findings, any subjects who were diagnosed with hyperlipidemia, CVA, CVD, or diabetes mellitus, or began using statins after their OB diagnosis were systematically precluded from our study cohort.

The ultimate study cohort encompassed 54,909 individuals (mean age: 52.3; standard deviation: 13.1), with a distribution of 29,828 (54.3%) females and 25,081 (45.7%) males. The baseline characteristics of the study subjects are thoroughly detailed in Table 1.

### Statistical Methods

Our study analyzed the association of various risk factors with OB, including CVA, CVD, gender, age, BMI, smoking habits, diabetes mellitus, hyperlipidemia, and statin use. Age and BMI were treated as continuous variables in the statistical models regarding them as risk factors, while being employed as categorical variables for the purposes of data balancing. Conversely, all other variables were dichotomized.

To elucidate the role of individual risk factors, we employed a two-stage analytic strategy. Initially, we utilized stabilized inverse probability of treatment weighting (IPTW) [19,20,21] to mitigate potential confounding and achieve balance in the data relative to each specific risk factor. Consequently, logistic regression models were constructed incorporating the corresponding weights and confining the set of predictors to the particular risk factor under investigation. This method produced odds ratios, indicating the magnitude of each risk factor’s impact. To account for multiple testing, we made necessary adjustments to the *p*-values through the Benjamini–Hochberg method [22], thereby maintaining control over the false discovery rate.

As an illustrative example of single variable analysis, let us consider age as the variable. After employing stabilized IPTW, we created a balance in our data distribution with respect to age, minimizing systematic differences between older and younger participants. Upon building a logistic regression model with age as the sole covariate, we were able to interpret the derived odds ratio as the increase (or decrease) in odds of OB associated with each unit increase in age.

Further in our analytical procedure, we capitalized on subgroup balancing propensity scores (SBPSs) [23] to discern the compound effect of a risk factor within specific strata of our dataset. Such subgroup analyses included evaluating the influence of age and BMI on male and female patients separately, the repercussions of age and BMI on patients with and without hyperlipidemia, and the bearing of hyperlipidemia on patients who were on or off statin therapy. For each of these sub-analyses, relative risk comparisons were graphically represented, with confidence intervals derived via bootstrap resampling [24].

An additional analysis was conducted focusing on the medications prescribed within the initial month following an OB diagnosis. For this investigation, the dataset excluded patients who had received medications before the start of this specific analysis and only included data from patients who had been prescribed a single medication, accounting for 86% of the OB patients. This analysis was purely descriptive in nature, involving no balancing of the data or estimation via a model.

Overall, our methodology was designed to systematically scrutinize the relationship between the selected risk factors and OB, using rigorous statistical techniques to ensure accuracy and reliability of our findings.

A power analysis was conducted to assess the study’s ability to detect an odds ratio of 1.1 for the association between olecranon bursitis and various risk factors. This analysis used a simulation-based approach tailored for logistic regression models, with a sample size of 54,909 participants, a significance level (alpha) of 0.05, and 1000 simulation iterations.

The power analysis indicated high power for gender (power = 99.3%), smoking (power = 98.9%), and hyperlipidemia (power = 83.2%), suggesting sufficient sensitivity to detect the hypothesized effect size for these variables. Moderate power was observed for statins (power = 76.2%), while low power was observed for diabetes (power = 35.2%), cerebrovascular accidents (CVAs; power = 14.9%), and cardiovascular disease (CVD; power = 24.2%), which also coincides with the large confidence intervals we observed for the risk factors with low power.

All computational and statistical analyses were carried out using R version 3.6.0 (R Project for Statistical Computing), predominantly utilizing the packages WeightIt and Cobalt [25].

## 3. Results

**Figure 2:**
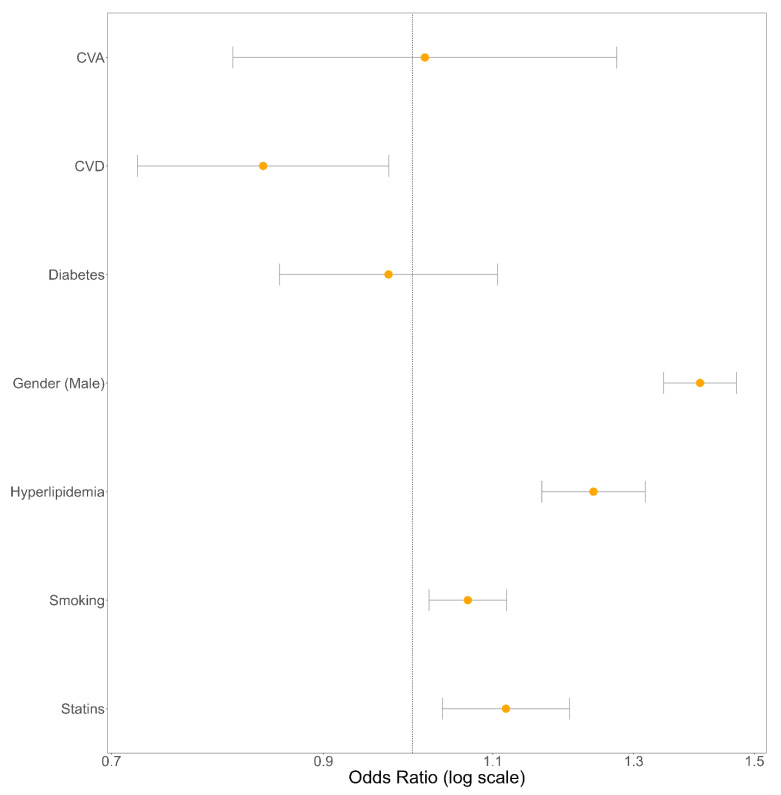
Forest plot illustrating the odds ratios for all binary risk factors associated with olecranon bursitis, estimated by the fully adjusted logistic regression model.

Our analysis found a pronounced link between gender and OB, with gender proving to be the most impactful risk factor (OR: 1.406; 95% CI: [1.347, 1.468]; *p*-value: <0.0001). Hyperlipidemia came next in line as a notable risk factor (OR: 1.243; 95% CI: [1.170, 1.322]; *p*-value: <0.0001). Other meaningful contributors, though having a small effect size, included statin use (OR: 1.130; 95% CI: [1.048, 1.218]; *p*-value: <0.003) and smoking habits (OR: 1.068; 95% CI: [1.020, 1.118]; *p*-value: <0.0086). Both age and BMI were modeled as continuous variables and showed significant effect sizes, discussed later when reviewing the relative risk across the ranges of age and BMI. The odds ratios for all binary risk factors are graphically displayed in Figure 2.

Nevertheless, some risk factors did not show any significant correlation with OB. In particular, CVA and diabetes mellitus were found to have no substantial link with OB, as denoted by their respective odds ratios and *p*-values shown in Table 2.

Visualizations of our efforts to achieve data balance via inverse probability of treatment weighting for each risk factor are provided in the Supplementary Material. It is worth noting that the model encountered difficulties in effectively balancing data concerning CVD as a risk factor. Hence, while we present the odds ratio for CVD for the sake of completeness, this estimate might be biased, and its significance is uncertain.

Our data analysis further enabled us to investigate the influence of risk factors within specific cohorts of interest. The successful balancing of these cohorts in terms of multiple risk factors is demonstrated in the Supplementary Material via the balancing plots using subgroup balancing propensity scores.

**Figure 3:**
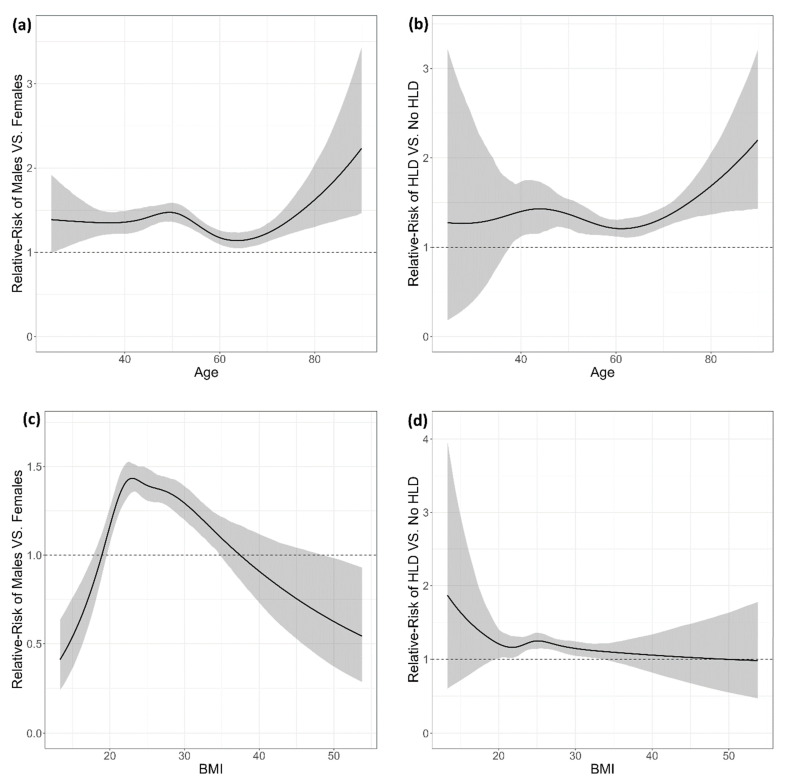
Relative risk (RR) plots illustrating the association between risk factors and olecranon bursitis (OB) stratified by gender, hyperlipidemia (HLD), age, and BMI. (**a**) RR of OB for males versus females across age groups. The risk is higher for males, particularly around age 50, with a slight decrease after age 60. (**b**) RR of OB in individuals with hyperlipidemia (HLD) versus those without, across age groups. (**c**) RR of OB for males versus females across BMI ranges. (**d**) RR of OB in individuals with hyperlipidemia (HLD) versus those without, across BMI ranges. Shaded regions represent 95% confidence intervals for each relative risk estimate.

Figure 3a visualizes the gender-related risk of OB across varying ages, revealing a persistently higher relative risk for males across all age brackets. The relative risk for males peaks around age 50, with males being 1.472 times more likely to develop OB (95% CI: 1.361, 1.588). However, this risk disparity diminishes around the age of 65, where the relative risk falls to 1.143 (95% CI: 1.051, 1.241). The influence of age as a risk factor was also analyzed for two subgroups: those with hyperlipidemia (HLD) and those without (NO HLD). As illustrated in Figure 3b, beginning at age 40, the presence of HLD amplifies the risk of OB, with the risk ranging from 1.395 (95% CI: 1.107, 1.722) around age 40 to 1.685 (95% CI: 1.367, 2.054) around age 80.

When analyzing BMI as a risk factor among male and female subgroups, the findings depicted in Figure 3c reveal that males have a higher risk of OB than females for BMI values between 20 and 35. The relative risk peaks around BMI 23, with a value of 1.433 (95% CI: 1.353, 1.520). Subsequent analysis considered BMI as the risk factor among HLD and non-HLD subgroups. As demonstrated in Figure 3d, the most significant relative risk of HLD was 1.247 (95% CI: 1.142, 1.358), which was identified around a BMI value of 25.

An analysis considering the use of statins as the risk factor was conducted with HLD and non-HLD patients as the subgroups. The analysis addressed the question of whether the effect of statins on the risk of OB differs when comparing two groups of patients: those with HLD and those without it. Our findings showed that the relative risk (RR) is quite similar in both groups: The RR of OB due to statins was 1.183 for the group of patients without HLD and 1.214 for the group of patients who did have HLD, and these relative risks had large overlapping 95% confidence intervals. In other words, we found no evidence that the effect of statins on the risk of OB is significantly different between patients with and without HLD, neither increasing nor decreasing their existing risk of OB in those groups.

**Figure 4:**
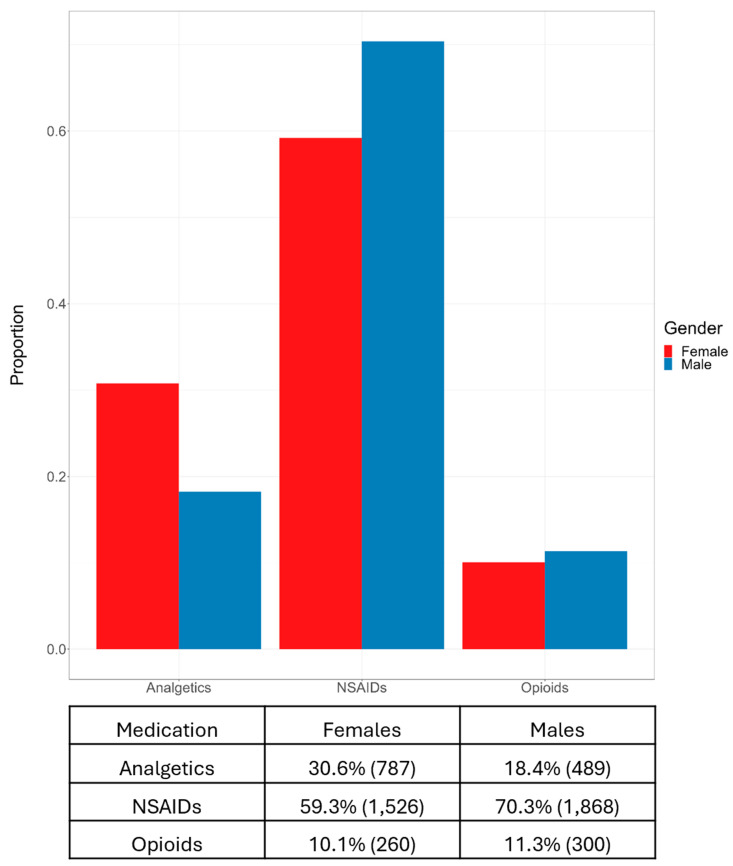
Proportion and frequency of medication usage by gender within the first month after an OB diagnosis, highlighting differences in the use of analgetics, NSAIDs, and opioids among females and males.

Supplementary analysis was undertaken, specifically targeting the medications administered within the first month subsequent to an OB diagnosis. The dataset utilized for this exploration deliberately excluded patients who had been administered medications prior to the initiation of this analysis. It solely incorporated data from patients prescribed a single medication, representing 86% of the OB patients. This examination was strictly descriptive, entailing no data balancing or model-based estimations, and its results are shown in Figure 4.

## 4. Discussion

The precise etiology of OB remains nebulous, likely being multifactorial in nature. Our population-based study encompassing over 10,000 patients has found a strong and significant effect of hyperlipidemia on olecranon bursitis. The odds of OB occurrence in patients with hyperlipidemia were 1.23 times higher than the odds of OB occurrence in patients without hyperlipidemia. Notably, the influence of age as a risk factor was also found to be compounded by the presence of hyperlipidemia, and beginning at age 40, the presence of hyperlipidemia was found to amplify the risk of OB. Hyperlipidemia is a systemic metabolic disorder marked by abnormal blood lipid levels, known for its impact on the vascular system and internal organs [12,26]. Recent studies found hyperlipidemia to have a significant impact on tendon disorders. Several pathophysiological pathways have been identified linking hyperlipidemia to certain tendon pathologies, such as rotator cuff tears [14,15,17,27] and adhesive capsulitis [12,13,28]. Tendons, with their high metabolic demands, rely on sufficient blood supply to deliver oxygen and nutrients [26]. In hyperlipidemic states, cholesterol byproducts accumulate, compromising blood flow to tendons and potentially impairing their physiological function, leading to fatigue and injury [15,16]. Cholesterol deposition also alters collagen synthesis, causing structural and biomechanical changes in the tendon matrix [17]. Hyperlipidemia, linked to systemic inflammation, activates the NF-κB pathway, increasing pro-inflammatory cytokines like TNF-α and IL-6 [17]. It also impairs tendon progenitor cells by suppressing tendon-related genes and inducing apoptosis and autophagy via ROS-activated NF-κB and AKT/FOXO1 pathways, leading to tendon degeneration and tendinopathy [15,17].

Our findings also indicate that statin therapy (Hydroxymethylglutaryl coenzyme A reductase inhibitors) increases the risk of OB diagnosis (OR: 1.117; 95% CI: [1.036, 1.205]). This is consistent with the known association between statin use and muscle-related symptoms, including tendinopathy and tendon ruptures [29,30,31]. A recent nationwide study by Kwak et al. found a significantly higher risk of various tendinopathies, including trigger finger, radial styloid tenosynovitis, elbow epicondylitis, rotator cuff tendinopathy, and Achilles tendinitis, in statin users, regardless of the statin type [29]. One proposed mechanism is that statins increase matrix metalloproteinase (MMP) production, such as MMP-1 and MMP-13, which weakens the tendon matrix [32].

The risk of OB in our study was consistently higher in males compared to females (OR: 1.406; 95% CI: [1.347, 1.468]; *p*-value: <0.0001). In males, the relative risk peaks around age 50 and declines by age 65. This increased risk was observed consistently in males with BMI values ranging from 20 to 35. These findings align with several previous studies that also demonstrated a male predominance [2,6,7,8], particularly in young and middle-aged men. Interestingly, one earlier study on a military population reported similar OB occurrence in males and females performing non-manual, administrative duties [3]. Another study observed a higher prevalence in females in rural Spain, attributed to the greater number of women engaged in manual labor [33]. Since our study did not account for patients’ occupations or history of prior trauma, the underlying reason for the observed male predominance in OB remains unclear.

Diabetes is associated with various musculoskeletal conditions, including osteoarthritis, adhesive capsulitis, carpal tunnel syndrome, trigger finger, plantar fasciitis, and rotator cuff tendinitis [34]. The underlying mechanisms remain unclear, but one hypothesis suggests that hyperglycemia induces collagen glycosylation and excessive accumulation of advanced glycation end-products (AGEs), resulting in collagen stiffness, impaired extracellular matrix function, and increased tissue fragility [35]. Another theory implicates AGE binding to tissue receptors, promoting inflammation and contributing to bursitis and tendinitis [36]. Several studies have proposed a link between diabetes and olecranon bursitis (OB) [2,4,5,10,37], but large-scale studies confirming this association are lacking. Importantly, our results suggest that the risk of olecranon bursitis is not significantly elevated in patients with diabetes mellitus. This may be attributed to our inability to differentiate between septic and non-septic cases of olecranon bursitis. Although earlier studies have proposed a potential association between diabetes and OB, these findings are largely derived from retrospective case series rather than robust large-scale epidemiological studies. Notably, two systematic reviews investigating the characteristics and treatment of olecranon bursitis reported that the majority of documented cases involve septic (infected) bursitis, which is more prevalent in immunocompromised individuals, including those with diabetes [7,38]. In our cohort, we were unable to differentiate between septic and aseptic bursitis. However, we hypothesize that most cases are aseptic, as the data were sourced from outpatient clinic records rather than hospitalizations or emergency department visits. Since aseptic bursitis has a weaker association with diabetes compared to septic bursitis, this may explain why diabetes did not emerge as a significant risk factor in our study population.

Our study has certain limitations. Firstly, as this was a retrospective study conducted on a large de-identified database and relying on diagnosis codes, our access to individual patient management and outcomes was restricted. Therefore, we could not ensure that standardized diagnostic criteria were consistently applied. However, OB is a straightforward condition typically diagnosed through clinical assessment. Consequently, we believe that the lack of specific diagnostic criteria does not compromise the validity of our study’s findings. Secondly, we were unable to distinguish between septic and aseptic olecranon bursitis, which are considered two distinct entities. Additionally, data on antecedent trauma and occupational or non-occupational exposure to biomechanical risk factors for tendinopathy were not collected. Despite a recent systematic review not finding any studies identifying links between OB and mechanical overload [39], occupational history was unavailable to us for analysis. In addition, OB is diagnosed clinically with a broad differential diagnosis. Lastly, the model struggled to balance data for CVD as a risk factor, so the reported odds ratio may be biased, and its significance is uncertain. However, given the nature of this population cohort study, we lacked the means to verify the accuracy of diagnoses made by physicians from various sub-specialties.

## 5. Conclusions

A comprehensive examination of a total of 54,909 individuals derived from a large-scale database revealed that the most significant risk factors for OB were gender, with males exhibiting a odds ratio of 1.406 vs. females for the likelihood of OB, and hyperlipidemia (OR: 1.243). Other notable contributors, albeit with smaller effect sizes, included statin use (OR: 1.130) and smoking habits (OR: 1.068). CVA and diabetes mellitus were not significantly correlated with OB. The impact of age and BMI were significant, with males showing a higher risk of OB across most age and BMI brackets, and the risk of OB increasing with the presence of hyperlipidemia from age 40. The influence of statin use on the risk of OB was found to be similar for both hyperlipidemic and non-hyperlipidemic patients.

This study highlights several critical factors influencing the risk of OB, with significant implications for clinical practice. The strong association between hyperlipidemia and OB underscores the importance of screening for metabolic disorders in patients presenting with OB, especially among those with concurrent risk factors such as obesity and advanced age. The observed elevated risk linked to statin use highlights the need for clinicians to evaluate musculoskeletal symptoms in hyperlipidemic patients undergoing lipid-lowering therapy. Additionally, the persistent male predominance and variable risk across BMI and age groups suggest that tailored prevention strategies might be beneficial, particularly for middle-aged males. Smoking, while modestly increasing risk, also remains an important modifiable risk factor. The findings support a comprehensive, multidisciplinary approach to managing OB, integrating metabolic and lifestyle interventions alongside traditional treatments such as NSAIDs and physical therapy. Further investigation into the mechanisms of hyperlipidemia-associated bursitis may refine these strategies and guide targeted therapies.

## Figures and Tables

**Table 1:** Baseline characteristics of the study population by subgroup stratified by outcome (OB).

Variable	Without Olecranon Bursitis (n = 44,608)	With Olecranon Bursitis (n = 10,301)	All Patients (n = 54,909)
Age group			
18–44	14,301 (32.1%)	3064 (29.7%)	17,365 (31.6%)
45–54	13,323 (29.9%)	3256 (31.6%)	16,579 (30.2%)
55–64	8554 (19.2%)	2295 (22.3%)	10,849 (19.8%)
65–74	5671 (12.7%)	1286 (12.5%)	6957 (12.7%)
75–90	2759 (6.2%)	400 (3.9%)	3159 (5.8%)
BMI group			
10–18.4	1911 (4.3%)	229 (2.2%)	2140 (3.9%)
18.5–24	22,810 (51.1%)	4830 (46.9%)	27,640 (50.3%)
25–29	13,583 (30.4%)	3607 (35.0%)	17,190 (31.3%)
30–34	4496 (10.1%)	1185 (11.5%)	5681 (10.3%)
35–55	1808 (4.1%)	450 (4.4%)	2258 (4.1%)
Gender			
Female	25,034 (56.1%)	4794 (46.5%)	29,828 (54.3%)
Male	19,574 (43.9%)	5507 (53.5%)	25,081 (45.7%)
Smoking			
No	31270 (70.1%)	6933 (67.3%)	38,203 (69.6%)
Yes	13,338 (29.9%)	3368 (32.7%)	16,706 (30.4%)
Hyperlipidemia			
No	39,032 (87.5%)	8643 (83.9%)	47,675 (86.8%)
Yes	5576 (12.5%)	1658 (16.1%)	7234 (13.2%)
Diabetes Mellitus			
No	43,256 (96.9%)	9951 (96.6%)	53,2071 (96.9%)
Yes	1352 (3.1%)	350 (3.4%)	1702 (3.1%)
CVA			
No	44,191 (99.1%)	10,204 (99.1%)	54,395 (99.1%)
Yes	417 (0.9%)	97 (0.9%)	514 (0.9%)
CVD			
No	43,662 (97.9%)	10,071 (97.8%)	53,734 (97.9%)
Yes	946 (2.1%)	230 (2.2%)	1175 (2.1%)
Statins			
No	40,388 (90.5%)	9096 (88.3%)	49,484 (90.1%)
Yes	4220 (9.5%)	1205 (11.7%)	5425 (9.9%)

**Table 2:** Odds ratio for OB, estimated separately for each risk factor. Alongside the 95% confidence intervals are *p*-values which were adjusted for multiple comparisons.

Variable	Odds Ratio	95% CI	Adjusted *p*-Value
Diabetes Mellitus			
No (reference)	-	-	-
Yes	0.979	(0.859, 1.115)	0.7491
Gender			
Female (reference)	-	-	-
Male	1.406	(1.347, 1.468)	<0.0001
Smoking			
No (reference)	-	-	-
Yes	1.068	(1.020, 1.118)	0.0086
Hyperlipidemia			
No (reference)	-	-	-
Yes	1.243	(1.170, 1.322)	<0.0001
Hypertension			
No (reference)	-	-	-
Yes	0.848	(0.773, 0.931)	0.0013
CVA			
No (reference)	-	-	-
Yes	1.064	(0.850, 1.332)	0.6705
CVD			
No (reference)	-	-	-
Yes	0.885	(0.763, 1.027)	0.1437
Statins			
No (reference)	-	-	-
Yes	1.130	(1.048, 1.218)	0.0030

## Data Availability

Data are contained within the article and Supplementary Materials.

## Supplementary Materials

The following supporting information can be downloaded at: https://www.mdpi.com/article/10.3390/jcm13247801/s1.
