# Supplementary material for: Risk Factors for the Development of Olecranon Bursitis—A Large-Scale Population-Based Study
Source: J Clin Med. 2024 Dec 20;13(24):7801. doi: 10.3390/jcm13247801 (PMC11728362; doi:10.3390/jcm13247801)
Supplement: Supplementary file 1 [file jcm-13-07801-s001.zip › jcm-3294466-supplementary.pdf]

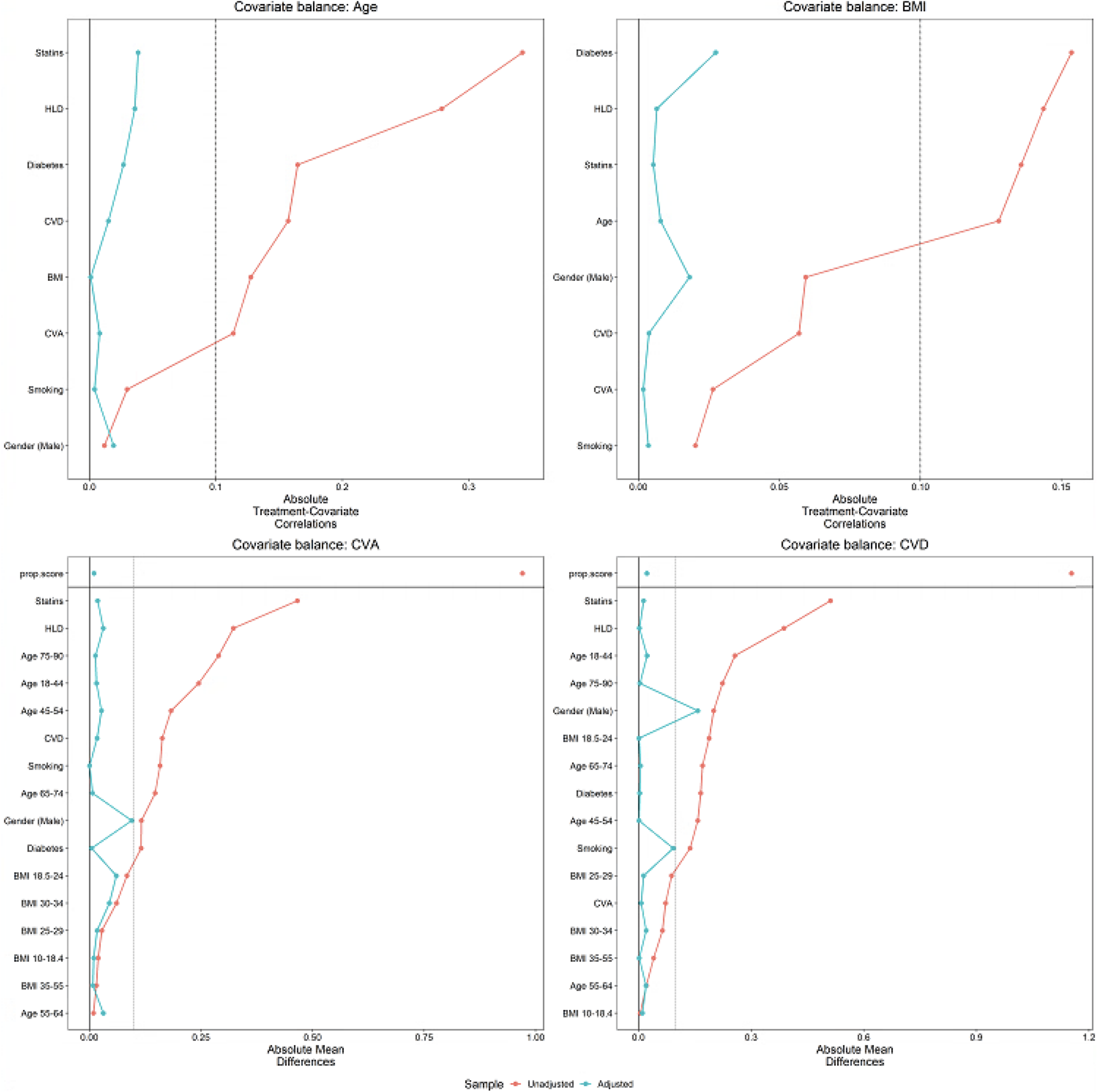
Covariate balance: Age
Statins
HLD
Diabetes
CVD
BMI
CVA
Smoking
Gender (Male)
0.0
0.1
0.2
0.3
Absolute
Treatment-Covariate
Correlations
Covariate balance: BMI
Diabetes
HLD
Statins
Age
Gender (Male)
CVD
CVA
Smoking
0.00
0.05
0.10
0.15
Absolute
Treatment-Covariate
Correlations
Covariate balance: CVA
prop.score
Statins
HLD
Age 75-90
Age 18-44
Age 45-54
CVD
Smoking
Age 65-74
Gender (Male)
Diabetes
BMI 18.5-24
BMI 30-34
BMI 25-29
BMI 10-18.4
BMI 35-55
Age 55-64
0.00
0.25
0.50
0.75
1.00
Absolute Mean
Differences
Covariate balance: CVD
prop.score
Statins
HLD
Age 18-44
Age 75-90
Gender (Male)
BMI 18.5-24
Age 65-74
Diabetes
Age 45-54
Smoking
BMI 25-29
CVA
BMI 30-34
BMI 35-55
Age 55-64
BMI 10-18.4
0.0
0.3
0.6
0.9
1.2
Absolute Mean
Differences
Sample
Unadjusted
Adjusted

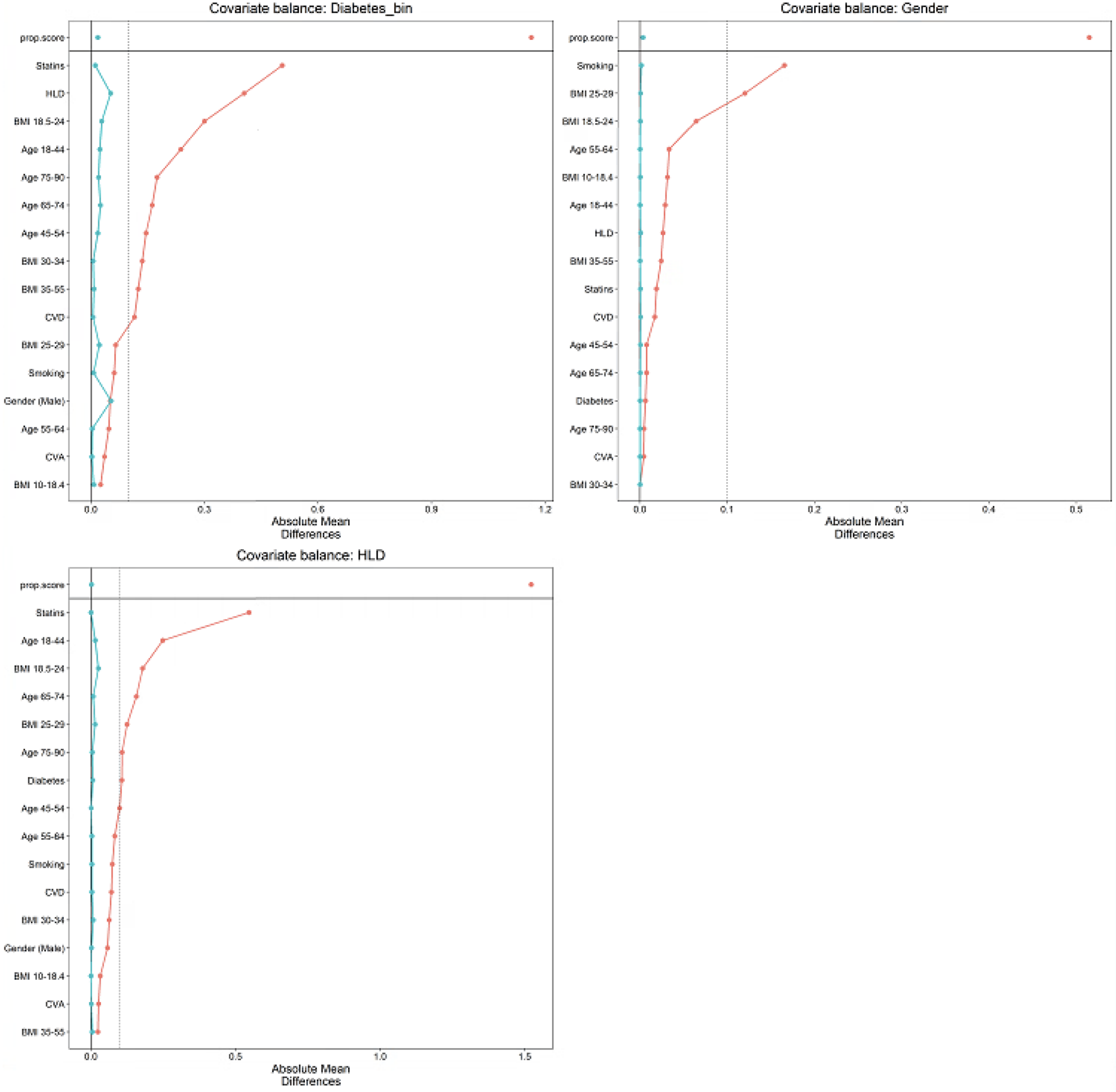
Covariate balance: Diabetes_bin
prop.score
Statins
HLD
BMI 18.5-24
Age 18-44
Age 75-90
Age 65-74
Age 45-54
BMI 30-34
BMI 35-55
CVD
BMI 25-29
Smoking
Gender (Male)
Age 55-64
CVA
BMI 10-18.4
0.0
0.3
0.6
0.9
1.2
Absolute Mean Differences
Covariate balance: Gender
prop.score
Smoking
BMI 25-29
BMI 18.5-24
Age 55-64
BMI 10-18.4
Age 18-44
HLD
BMI 35-55
Statins
CVD
Age 45-54
Age 65-74
Diabetes
Age 75-90
CVA
BMI 30-34
0.0
0.1
0.2
0.3
0.4
0.5
Absolute Mean Differences
Covariate balance: HLD
prop.score
Statins
Age 18-44
BMI 18.5-24
Age 65-74
BMI 25-29
Age 75-90
Diabetes
Age 45-54
Age 55-64
Smoking
CVD
BMI 30-34
Gender (Male)
BMI 10-18.4
CVA
BMI 35-55
0.0
0.5
1.0
1.5
Absolute Mean Differences

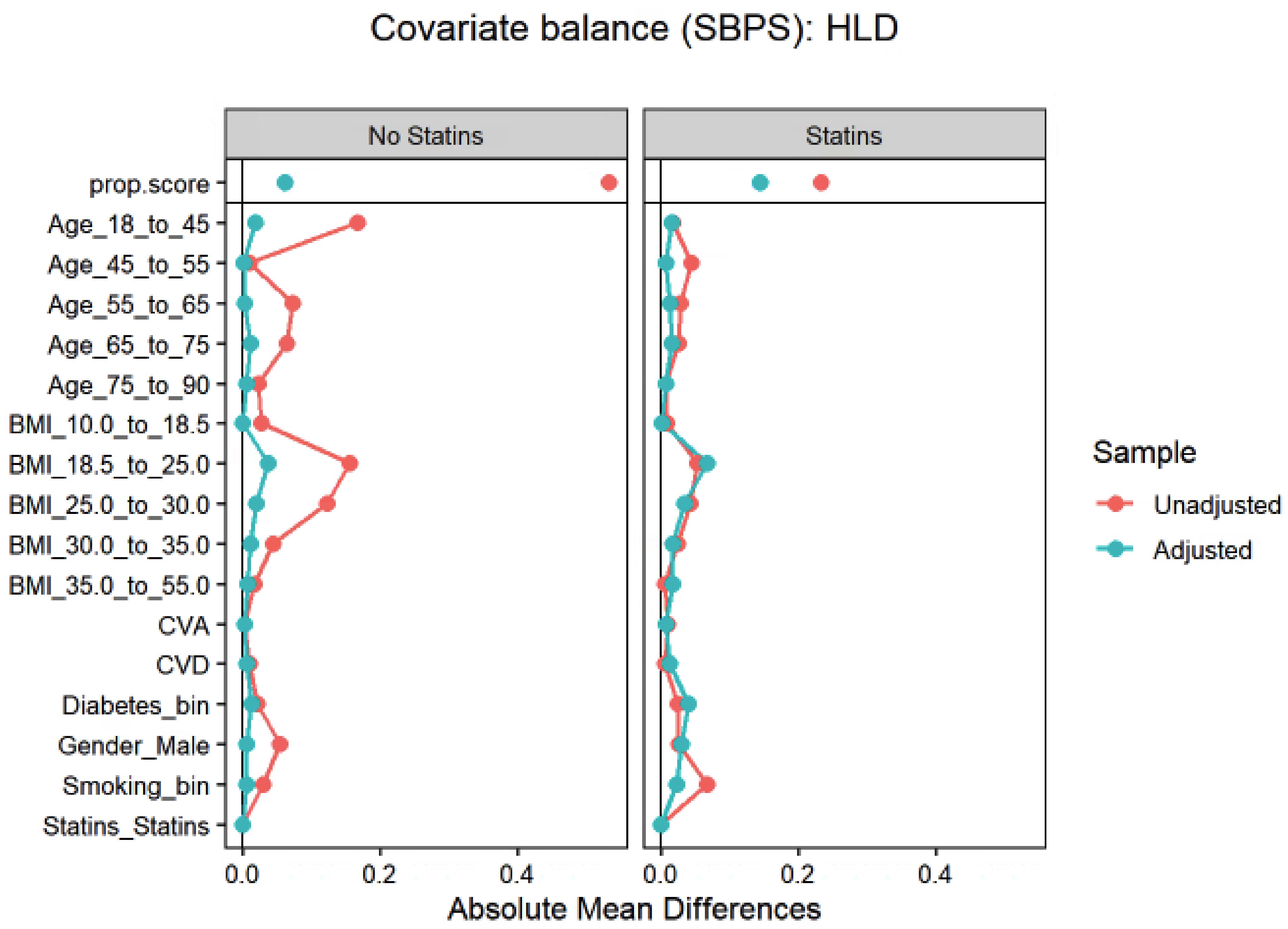
Covariate balance (SBPS): HLD
No Statins
Statins
prop.score
Age_18_to_45
Age_45_to_55
Age_55_to_65
Age_65_to_75
Age_75_to_90
BMI_10.0_to_18.5
BMI_18.5_to_25.0
BMI_25.0_to_30.0
BMI_30.0_to_35.0
BMI_35.0_to_55.0
CVA
CVD
Diabetes_bin
Gender_Male
Smoking_bin
Statins_Statins
0.0
0.2
0.4
0.0
0.2
0.4
Absolute Mean Differences
Sample
Unadjusted
Adjusted

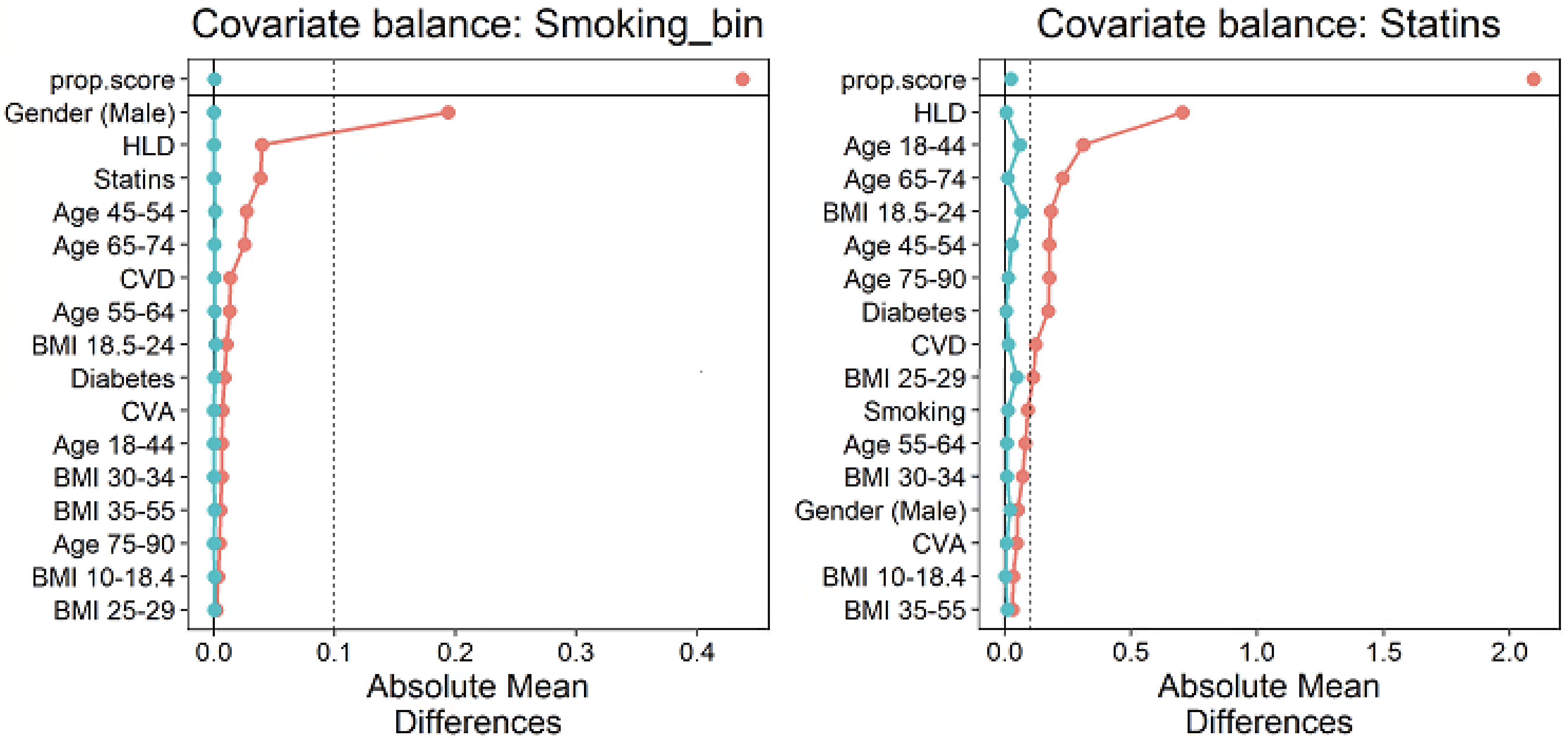
Covariate balance: Smoking_bin
prop.score
Gender (Male)
HLD
Statins
Age 45-54
Age 65-74
CVD
Age 55-64
BMI 18.5-24
Diabetes
CVA
Age 18-44
BMI 30-34
BMI 35-55
Age 75-90
BMI 10-18.4
BMI 25-29
0.0
0.1
0.2
0.3
0.4
Absolute Mean Differences
Covariate balance: Statins
prop.score
HLD
Age 18-44
Age 65-74
BMI 18.5-24
Age 45-54
Age 75-90
Diabetes
CVD
BMI 25-29
Smoking
Age 55-64
BMI 30-34
Gender (Male)
CVA
BMI 10-18.4
BMI 35-55
0.0
0.5
1.0
1.5
2.0
Absolute Mean Differences

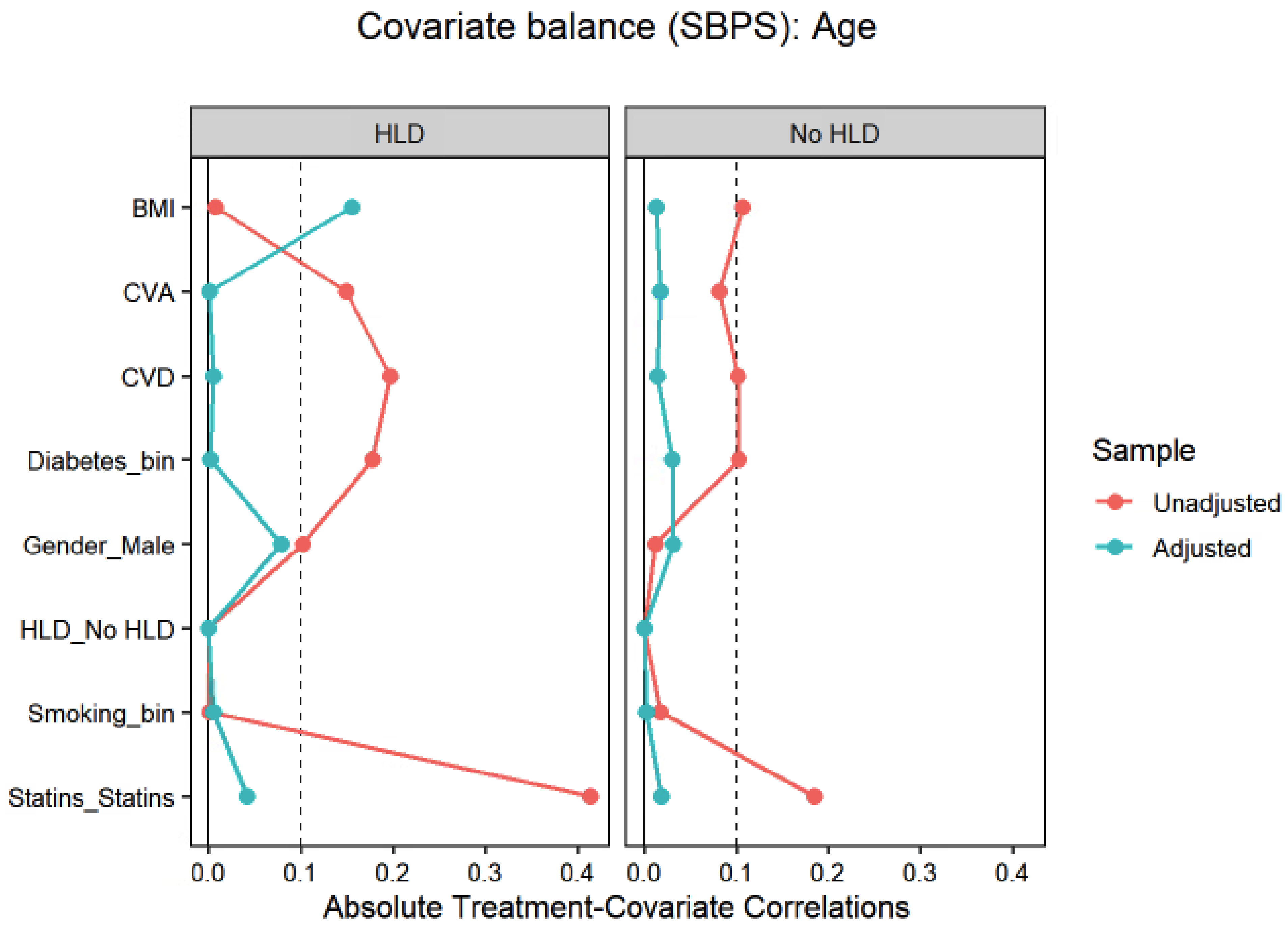

Covariate balance (SBPS): Age
HLD
No HLD
BMI
CVA
CVD
Diabetes_bin
Gender_Male
HLD_No HLD
Smoking_bin
Statins_Statins
0.0
0.1
0.2
0.3
0.4
Absolute Treatment-Covariate Correlations
Sample
Unadjusted
Adjusted

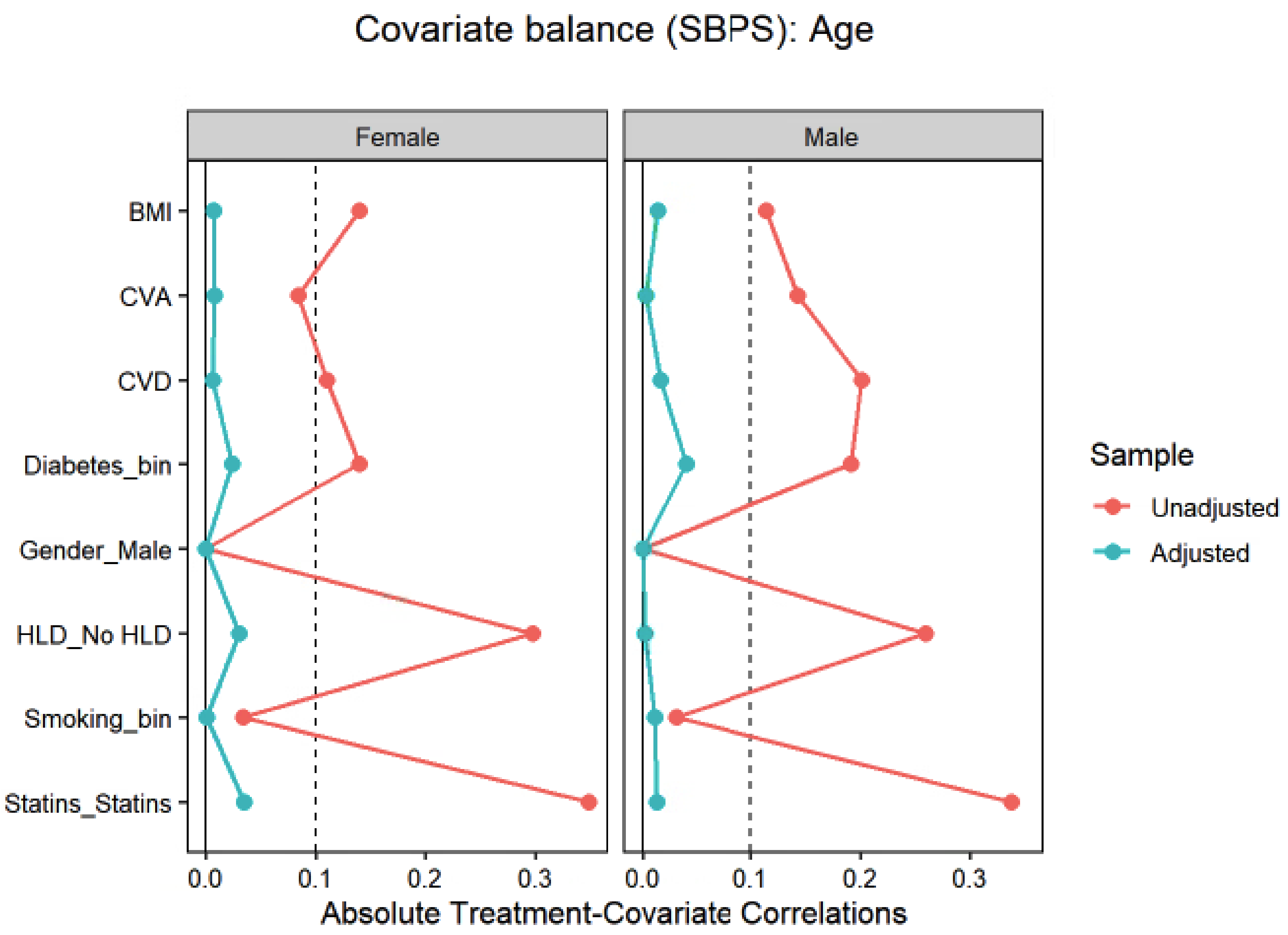

Covariate balance (SBPS): Age
Female
Male
BMI
CVA
CVD
Diabetes_bin
Gender_Male
HLD_No HLD
Smoking_bin
Statins_Statins
0.0
0.1
0.2
0.3
Absolute Treatment-Covariate Correlations
Sample
Unadjusted
Adjusted

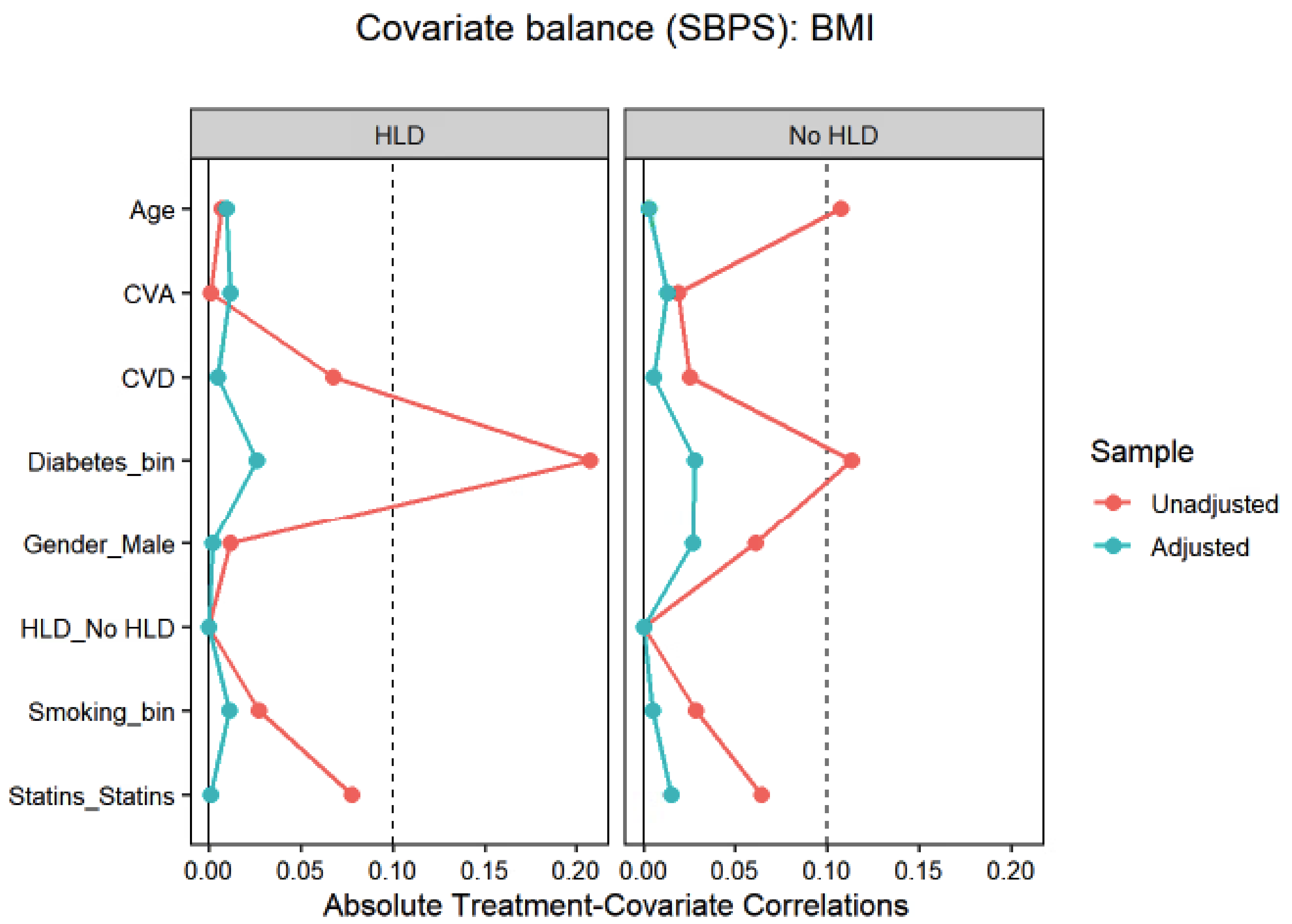
Covariate balance (SBPS): BMI
HLD
No HLD
Age
CVA
CVD
Diabetes_bin
Gender_Male
HLD_No HLD
Smoking_bin
Statins_Statins
0.00
0.05
0.10
0.15
0.20
Absolute Treatment-Covariate Correlations
Sample
Unadjusted
Adjusted

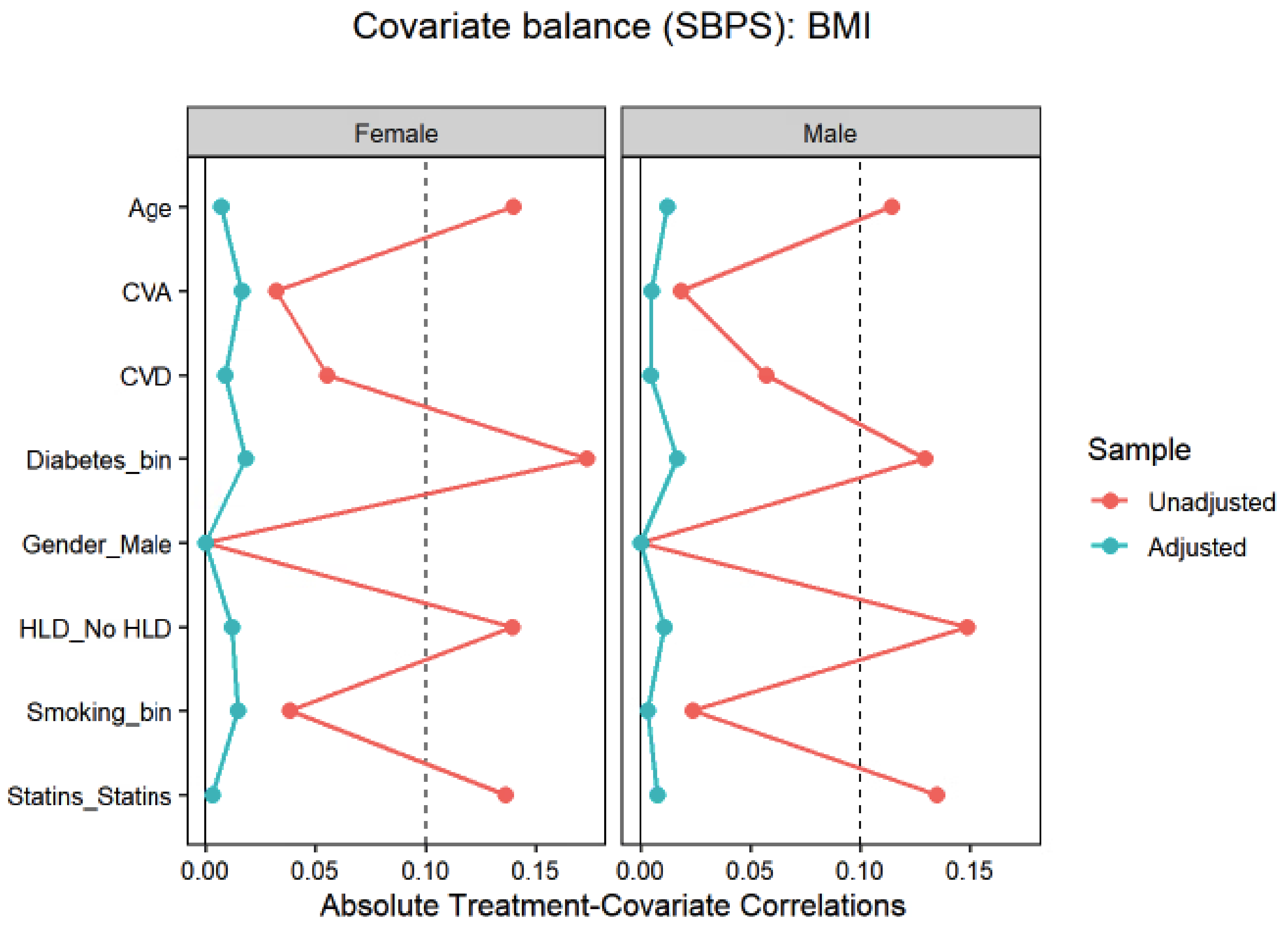
Covariate balance (SBPS): BMI
Female
Male
Age
CVA
CVD
Diabetes_bin
Gender_Male
HLD_No HLD
Smoking_bin
Statins_Statins
0.00
0.05
0.10
0.15
Absolute Treatment-Covariate Correlations
Sample
Unadjusted
Adjusted
